# Resilience, Sense of Coherence, and Coping with Stress as Predictors of Psychological Well-Being in the Course of Schizophrenia. The Study Design

**DOI:** 10.3390/ijerph16071266

**Published:** 2019-04-09

**Authors:** Bernadetta Izydorczyk, Katarzyna Sitnik-Warchulska, Aleksandra Kühn-Dymecka, Sebastian Lizińczyk

**Affiliations:** 1 Institute of Applied Psychology, Faculty of Management and Social Communication, Jagiellonian University in Krakow, Krakow 30-348, Poland; 2 Institute of Psychiatry and Neurology, Warsaw 02-957, Poland; a.a.dymecka@gmail.com; 3 Katowice Faculty of Psychology, SWPS University of Social Sciences and Humanities, Katowice 40-326, Poland; octans@wp.pl

**Keywords:** schizophrenia, resilience, sense of coherence, coping with stress, psychological well-being, risk factors

## Abstract

The main research objective was seeking for the predictive role of such personal resources as resilience, sense of coherence, and coping with stress in psychological well-being of schizophrenia patients and their treatment. The study group comprised 201 individuals with schizophrenia, aged between 18 and 62. The following instruments were used: The sense of coherence scale SOC-29, The resilience scale for adults, polish adaptation of the stress appraisal measure, semistructured clinical interview, the positive and negative syndrome scale, the mood scale, and the general health questionnaire. A stepwise regression analysis aimed at selecting a group of significant predictors for the verified factors of psychological well-being in patients suffering from schizophrenia was carried out. The results of the study demonstrated the following to be significant predictors of psychological well-being in patients with schizophrenia: Resilience (explaining significantly the level of schizophrenic symptomatology Beta = −0.30, negative symptoms Beta = −0.385, and cognitive disorders Beta = −0.303), sense of coherence, which significantly predicted mood (in the case of manageability, Beta = 0.580 for positive mood, and Beta = 0.534 for negative mood) and psychiatric symptomatology (comprehensibility, Beta = 0.311 for negative symptoms, Beta = 0.173 for excessive arousal, and Beta = 0.330 for cognitive disorganization). The level of perceived stress appraised as challenge predicted positive mood (Beta = 0.164), while stress appraisal in terms of threat served as a predictor for negative mood and depressiveness (Beta = 0.190). The study results can prove helpful in creating therapeutic and programs and psychiatric rehabilitation for patients with schizophrenia.

## 1. Introduction

According to literature, it is assumed that various predictors may be significant for a positive course of schizophrenia, up to a patient’s recovery [1,2,3]. Psychosocial resources and deficits or defects of biological and psychosocial structures are important factors that determine the status of psychological well-being as well as course of the disease and the process of recovering from schizophrenia [1,2]. However, no Polish studies strived to verify simultaneously the predictive role of a set of three interrelated personal resources directed at protecting ego strength, that is: Resilience, sense of coherence, and perceiving stress as a threat versus challenge within a group of patients with schizophrenia similar in terms of sociodemographic variables.

The mentioned resources play a significant role in the process of treating patients with schizophrenia. Contemporary theories explaining the multilayer nature of schizophrenia background emphasize the role of the strength of interaction between disease predispositions and factors causing psychological stress in the course of developing symptoms of psychosis, developing relapses, and exacerbations of the psychosis [1]. The study by Birchwood and Jackson [1] (2006) indicates that the course of schizophrenia and the psychophysical condition of patients may be highly diverse: 20% to 25% of patients with schizophrenia experience clinical and social recovery. As Nuechterlein and colleagues [4] note, the intensification of risk factors (stressors) or attenuation of protective factors (i.e., psychological potential) in the course of schizophrenia may be a vital source of transitioning from remission to prodromic states; obviously, the significance of individual differences between patients with schizophrenia must also be included in this context. Many long-term studies and observations of patients diagnosed with schizophrenia indicate that individuals deal with the tough experience of severe mental disorder in a number of ways: Around 25–30% of the patients can be considered as cured, while 25–45% of them as individuals who have improved significantly, which includes relatively independent functioning in a given role [5,6,7].

Seeking an answer to the question why an individual “moves” towards health along the health–illness dimension, Antonovsky [8] defined the constructs of generalized resistance resources (GRR) and sense of coherence (SOC). Both resilience and sense of coherence as well as the level of perceived stress are psychological variables that significantly support the level of psychological well-being of an individual functioning in the situation of a chronic mental disorder [9,10].

Resilience is a person’s individual characteristic [11,12], a psychological variable describing the level of mental toughness of an individual, involving the ability to recover (adapt) from difficult experiences to one’s life situation [13,14]. The following attributes most commonly define resilience in studies: Self-efficacy [15], hope [15,16], and coping [11,15]. The following consequences of resilience were identified: Physical or psychological integration [11,15,16,17], developing personal control [15,18,19], psychological adjustment [15,18], and personal development in response to shock [11,15,19]. In their study, Torgalsbøen and Rund [20] emphasized that the ability to combat the disease as well as recovery after numerous years of experiencing symptoms of schizophrenia should be regarded in terms of combining the experience of disease symptoms with a high level of resilience. Deegan’s study of patients with schizophrenic disorders revealed that half of the sample improved their health state significantly [21]. Friborg and colleagues examined subjects with mental disorders and people with no such disorders [22]. It was noted that resilient people, who overcome difficult life conditions, are rather flexible than sensitive and use both their own and environmental protective resources while coping with difficulties. According to the latest, cross-sectional study by Rossi et al. [23], low resilience is directly associated with depression severity in patients with schizophrenia. Palmer et al. [24] also investigated the construct of resilience in the patients with schizophrenia. Compared to healthy controls, patients suffering from schizophrenia showed a lower level of resilience. However, higher levels of happiness were associated with higher levels of resilience, higher levels of optimism, and lower levels of perceived stress. Mizuno and colleagues [25] emphasized that resilience is the multidimensional concept, and there is a lack of standards in assessing resilience in schizophrenia.

Another personal resource significant for the recovery of patients with schizophrenia is sense of coherence. Sense of coherence plays the role of a buffer that limits experiencing negative emotions and pathology in the forms of depressiveness and anxiety [26]. According to Świtaj and colleagues [27] the sense of coherence is an important, personal resource that is correlated with quality of life in patients with mental illnesses. In the analysis conducted by Witkowska-Łuć [28], a higher level of sense of coherence, especially sense of comprehensibility, was found to be associated with a lower intensity of negative symptoms and overall psychiatric symptoms in the positive and negative symptoms scale (PANSS) during hospital admission.

Through highlighting individual differences in response to stress, research concerning resilience shed a new light on the general results of studies on vulnerability [29,30]. According to the stress appraisal theory by Lazarus and Folkman [31], two cognitive processes are related to coping stress: Primary appraisal and secondary appraisal. The cognitive assessment of the stressor (primary appraisal) is particularly important. The primary appraisal involves determining whether the event is stressful and whether the stressor poses a threat, or causes loss/ harm, or presents a challenge. The primary assessment determines the intensity and type of emotional response to the stressor. The loss/harm will trigger hostility, sorrow, sadness; the threat will be accompanied by fear, anxiety, and worry, and the challenge will cause both positive and negative emotions. Facing a stressor, the secondary appraisal follows, which involves the individual’s evaluation of the resources. Kang and colleagues [32] indicate that coping styles in individuals at high risk for psychosis are significantly associated with their cognitive appraisal of stress. Their study in individuals at ultra-high risk for psychosis and healthy controls revealed that the lower the hostility bias, the greater the tendency to use problem-focused forms of coping. In turn, the tendency to blame others for stressful situations was significantly associated with more passive and emotional forms of coping (tension-reduction) [32]. As Rüsh et al. [33] noted, a higher distress score may be associated with a stronger illness identity (experience of symptoms), and more perceived harm due to stigma among people at risk of psychosis may be a predictor for transition to schizophrenia. However, there is a lack of research describing the link between the psychological well-being in patients suffering from schizophrenia and the cognitive appraisal of stress.

In summary, a psychological diagnosis of personal resources’ structure that involves measuring one’s sense of coherence and resilience and perceived stress (assessing the protective factor), as well as adjusting the therapeutic intervention and psychological help to the obtained results, may be significant for the efficiency of recovery and the course of psychiatric rehabilitation. A therapeutic approach that incorporates the outcome of psychological diagnosis that involves continuous monitoring of the level of resilience, sense of coherence, and coping with stress is in line with contemporary standards of treating and rehabilitating patients with schizophrenia. It is worthwhile to note the high significance of this issue in the process of the recovery of a significant group of patients with schizophrenia in young age, who experience a need to develop numerous roles and sociovocational functions in their environment and everyday life. Seeking predictors (psychological resources—i.e., correlates of health) was the task of the authors of this article.

The main research objective of this study was seeking for the predictive role of such personal resources as resilience, sense of coherence, and coping with stress in psychological well-being of schizophrenia patients and their treatment. The assessment concerned the strength of the relationship between resilience, sense of coherence, level of perceived stress, and the level of psychological well-being in subjects with schizophrenia.

Within the research model, the dependent (explained) variable was labeled as “psychological well-being” in patients suffering from schizophrenia. The variable was defined as a theoretical construct describing the level of the general psychological condition of an individual suffering from schizophrenia, which was diagnosed and documented as requiring treatment. The variable was verified through measuring the following indices: Index of disease course (i.e., quotient of the number of hospitalizations in relation to the number of years of disease), index of psychiatric symptomatology (level of current disease symptoms: Negative and positive, depressiveness, anxiety, cognitive disorders), general level of mental health (psychological condition) in patients with schizophrenia, and the measurement of the current level of positive/negative mood.

The independent (explanatory) variable was defined as a collection of the following psychological resources: Resilience, sense of coherence, and level of perceived stress (appraisal as a challenge or threat), supporting the adaptational characteristics and psychological well-being in patients with schizophrenia. Within the assumptions of Antonovsky’s concept of salutogenesis [8], sense of coherence was defined as a general and complex human orientation describing the level of a schizophrenic patient’s conviction concerning the following: Information originating from both the external and internal environment is structured, predictable, and comprehensible, and the individual’s personal resources engage them in actions and allow coping with the requirements of everyday life in the course of chronic disease. The definition of sense of coherence adopted for the purposes of the study involves three indicators: Comprehensibility (which indicates the degree to which reality seems to be graspable and explainable), manageability (describing the level to which the individual possesses resources for managing the requirements of the arriving stimuli), and meaningfulness (describing the degree to which the individual emotionally feels that life makes sense and problems and requirements brought by it are worth effort, devotion, and engagement, rather than being solely a burden).

Resilience is a variable that describes the level of predicting and positive adjustment on various levels (intrapersonal and interpersonal) to life situations by an individual with schizophrenia, in spite of numerous risk factors and stressors [22].

The level of the perceived stress, according to Lazarus and Folkman’s concept [31], was defined as a third explanatory variable, entailing an individual’s generalized cognitive assessment concerning the experienced stress in terms of challenge or threat, burdening and exceeding their resources and threatening their well-being.

The sociodemographic variables were also controlled.

The following research questions were formulated:

- Does resilience explain psychological well-being in patients with schizophrenia, and to what degree?
- Does sense of coherence explain psychological well-being in patients suffering from schizophrenia, and to what degree?
- Does the level of perceived stress (as a challenge versus threat) in subjects suffering from schizophrenia explain psychological well-being, and to what degree?

## 2. Materials and Methods

### 2.1. Procedure and Study Population

The cross-sectional study was conducted in the years 2013–2017 in three psychiatric rehabilitation wards situated in a psychiatric clinic that gathered patients from across Poland. The purpose sampling method was adopted. A total of 250 patients with schizophrenia aged between 18 and 62 participated.

The following criteria for sample selection were adopted: Medical diagnosis of schizophrenia (F20-ICD10) with a relatively stable mental state that enabled a reliable completion of a set of questionnaires. Excluded from the study were patients with a co-existing addiction to psychoactive substances or other mental disorders. A group of 250 subjects diagnosed with paranoid schizophrenia (ICD10: F20.0), catatonic schizophrenia (F20.1), and undifferentiated schizophrenia (F20.3) were subjected with the research instruments.

All the subjects were patients of psychiatric rehabilitation wards. They received comprehensive information regarding the purpose, course, and terms of participation and signed an informed consent form. All subjects were informed about the voluntary nature of participating in the study and confidentiality of the obtained data. The procedures that were carried out in hospitals took place in each case after obtaining consent from the medical personnel. All the research procedures were performed in line with the 1964 Declaration of Helsinki with further amendments concerning studies with human participants. The Bioethics Board also approved the study.

In order to increase the reliability of the answers given and to create opportunity to explain potential doubts, subjects filled out the questionnaires in the presence of a research team member. Individuals who resigned from participation in the study during the course of completing the questionnaire were excluded from the study. As a result of that, the procedure was completed by 201 subjects (95 females and 106 males) aged between 18 and 54, diagnosed with paranoid schizophrenia.

### 2.2. Methods

The following instruments were used for measuring the independent variable:

- The sense of coherence scale (SOC-29) by Antonovsky (2005) in its authorized Polish version by the IPN Clinical Psychology Unit, the Unit of Psychoprophylaxis of the UAM Institute of Psychology, and the Occupational Psychology Unit at the Institute of Occupational Medicine in Łódź [34]. The questionnaire comprises 29 items that take the form of questions measuring sense of coherence and its three components of comprehensibility, manageability, and meaningfulness against a 7-point scale. It is possible to obtain results between 29 and 203 points. A high SOC-29 result denotes a strong sense of coherence. The following results for each subscale can be treated as high: For comprehensibility—11 to 77, for manageability (MA)—10 to 70, and for meaningfulness (ME)—8 to 56 points. The Cronbach α is 0.88.
- The resilience scale for adults (RSA) by Friborg et al. (2003), which serves the purpose of assessing resilience [22]. It comprises 6 factors measuring the following aspects: (1) perception of self, α = 0.70, (2) perception of future, α = 0.66, (3) *social competence*, α = 0.76, (4) structured style, α = 0.69, (5) family cohesion, α = 0.78, and social resources, α = 0.69. The mentioned RSA scales allow to measure protective factors describing the psychological characteristics labeled as resilience. The authors of this work used the Polish adaptation of RSA prepared by Jelonkiewicz, Kühn-Dymecka, and Zwoliński [35]. The Polish RSA scale comprises 20 items that measure personal strength (self-perception and perception of one’s own future), structured style, social competence, and the general factor. Subjects rated resilience responding to the RSA items on 5-point frequency scales (from: “I know how to accomplish” to “I am not sure”). The reliability of the whole scale is Cronbach α = 0.77, while the reliabilities of each of the subscales are as follows: For personal strength, Cronbach α = 0.74; for self-perception, α = 0.64; for perception of own future, α = 0.69; for social competences, α = 0.77; for structured style, α = 0.49. The internal consistency of the Polish RSA adaptation is highly similar to the original version. The only doubtful issue is the structured style scale, which demonstrated a much lower reliability than the original version [35]. Due to the unsatisfactory psychometric properties, total scores in the structured style scale were not used in the analyses.
- The stress appraisal measure for adolescents by Rowley et al. [36] in its Polish adaptation for adolescents and adults by Kosińska-Dec and Zwoliński [35] was used to measure the level of perceived stress. The instrument comprises 14 items for measuring general cognitive appraisal of the stress experienced by an individual in terms of challenge and threat. The scale for stress appraisal in terms of Challenge comprises 7 items (4 original ones and 3 created by the authors of the adaptation), and the threat appraisal scale consists of 7 original items. The whole instrument has demonstrated good internal consistency for both scales (Cronbach α for the challenge scale = 0.85, while for the threat scale, α = 0.82).

The following instruments were used to measure the dependent variable of psychological well-being:

- The positive and negative syndrome scale (PANSS) by Kay, Opler, and Fiszbein [37]. The PANSS scale consists of 30 items (number of obtainable points ranges between 30 and 210). The scale comprises 30 items divided into 3 parts: The first one (7 items) is designed to assess positive symptoms, the second one (7 items) negative symptoms, while the third one (16 items) the so-called general psychopathology. The instrument allows a clinical practitioner to assess positive, negative, and general symptoms as well as to calculate a total score. High scores indicate the level of psychopathological symptoms’ intensity. Total results that are lower than 60 points indicate progression in terms of symptoms, while results higher than 60 points indicate exacerbation of schizophrenic disorders. Each of the symptoms is assessed by the subjects against a 7-point scale. The instrument demonstrated good reliability (Cronbach α = 0.85).
- The mood scale by Wojciszke and Baryła [38], in a modified version by Jelonkiewicz, Kühn-Dymecka and Zwoliński [35]. The instrument consists of 20 items describing positive and negative mood. It measures positive and negative mood and the proportion of positive to negative mood. Subjects indicate the experienced feelings against 7-point frequency scales (from never to always). The scale has demonstrated good reliability, both for the positive mood scale (Cronbach *α* = 0.86) and negative mood scale (Cronbach *α* = 0.89).
- The general health questionnaire (GHQ-12) by Goldberg, in its Polish adaptation by Makowska and Merecz [39]. The general score allowed a description of the sense of mental health (psychological condition) in subjects. The scale comprises 12 items, including 6 ones concerning a description of one’s general psychological condition, while the remaining ones describe the level of positive health—i.e., level of concentration, feelings of happiness and playing a useful part, the ability to face problems, make decisions, and enjoy everyday activities. Subjects indicated the experienced changes against 4-point frequency scales (ranging from “not at all” to “much more than usual”). The Polish adaptation of GHQ demonstrated sufficient reliability (Cronbach *α* = 0.87) and validity confirmed through intergroup comparisons and factor analyses of versions of [36].

Semistructured clinical interviews were conducted to collect demographic characteristics (age, sex, level of education, source of income, living conditions, parent’s education, and family situation) and the following clinical variables: Age of symptom onset, year of first hospitalization, number of psychiatric hospitalizations, and duration of psychosis in years. The interviews were conducted by an experienced clinical psychologist (one of the authors of the study).

### 2.3. Research Model

**Figure 1:**
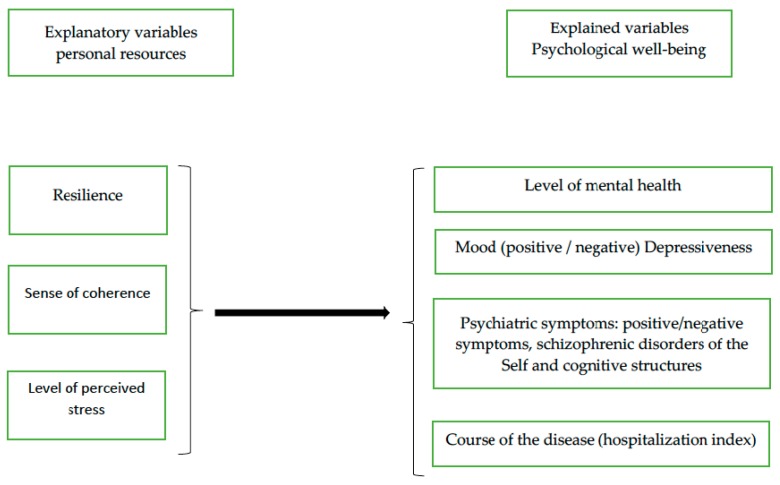
The research model: Personal resources as potential predictors of psychological well-being in patients with schizophrenia (own work).

The research model is presented in Figure 1. The presented research model is of a correlational-regression nature. It was assumed that the variables connected with personal resources significantly influence four dimensions of psychological well-being (level of mental health, mood, psychiatric symptoms, and hospitalization index) in the course of schizophrenia.

### 2.4. Statistical analysis

The results of the study were analyzed using the STATISTICA 8 (StatSoft, Kraków, Poland) and SPSS PC ver. 19.0 PL software (SPSS Inc. Chicago, IL, USA). Numeric and percentage frequencies as well as mean values and standard deviations were used for descriptive purposes. The Kolmogorov–Smirnov test was used to verify the normality of the results’ distribution. The criterion for statistical significance adopted in all calculations was *p* < 0.05.

At the first stage of describing the results, a descriptive portrayal of the sample was prepared through calculating means and standard deviations of all the variables. The second stage involved performing a stepwise regression analysis aimed at selecting a group of significant predictors for the verified factors of psychological well-being in patients suffering from schizophrenia and remaining in treatment for numerous years.

## 3. Results

A total of 92.5% of the subjects were diagnosed with paranoid complex, while the remaining ones suffered from catatonic (F20.1) or undifferentiated schizophrenia (F20.3). The mean age of becoming ill was 21.6 (range: 17–40) and the mean duration of the disease was 10 years (range: 1–41). The mean number of hospitalizations was almost 5 (range: 1–40).

Table 1 presents indices of the sociodemographic variables and the explained variable, i.e., psychological well-being in patients suffering from schizophrenia. The percentage of males was slightly higher, while 20.9% of the subjects received annuities. Most of the study participants completed high school (53.7) and higher education (35.3), while 73.6% of the subjects lived with their parent(s) and had not autonomized. The data presented in Table 1 reveal that the means for each of the scales used for measuring components of psychological well-being were situated in proximity of the middle of the scales’ potential range: For GHQ—12–2.32 points (range: 1.0–4.0), for PANSS—3.52 (range: 1.93–7.0), and for the mood scale: Negative mood—3.91 points (range: 1.0–7.0).

The profile of psychiatric symptoms based on the PANSS scale indicates that in the group of patients with schizophrenia, the most prominent symptoms were the following: Depression, conceptual disorganization, and negative symptoms. The following symptoms were lower in intensity: Anxiety, positive symptoms, and dysphoria. The data indicate a lack of high intensity of negative symptoms and depression in the sample at the point of conducting the study. However, the mean intensity of depression and negative symptoms qualified as a minimal and mild level of symptoms. The results of mean mood intensity in the sample indicate that the patients demonstrated similar frequencies of experiencing positive and negative mood when self-assessing their psychological condition using the mood scale. Results of the study point that exactly half of the subjects defined their own mood as positive, while the other half, conversely, declared their experienced mood to be negative.

Mean intensities of the components of the explained variable (i.e., resilience, sense of coherence, and level of perceived stress) are presented in Table 2.

As is demonstrated by the mean value for general resilience (63.01 out of 100 points), the subjects revealed a low level of global mental toughness. Compared with the maximum scores that can be obtained, subjects scored highest in mean resilience defined as personal strength (30.38 out of 50 points) and perception of self (17.72 points out of 30). The lowest mean score was noted for [erception of future (12.72 points out of 203). Out of the three verified factors describing coherence, meaningfulness proved to be higher than the others (comprehensibility and manageability), whose mean scores were on a similar level (42 points out of 70 for comprehensibility and 42.83 out of 72 for manageability). For each of the examined factors, the mean scores suggest a low intensity of sense of coherence in the sample. The obtained results also suggest that the subjects appraised the level of perceived stress experienced in the situation of the disease more frequently in terms of threat rather than a challenge, while the level of general coping with stress was demonstrated to be low (Table 2).

The first stage of the statistical analysis allowed the research team to determine mean levels of all variables. The mean results indicated low levels of both sense of coherence and resilience, as well as poor coping with stress (appraised as threat). In the second stage of the analysis, the stepwise progressive regression model was adopted. It served as a basis for verifying and identifying variables, which best explained the level of well-being in patients with schizophrenia. Due to the multitude of the statistical data obtained, Table 3 presents only the identified significant predictors (resilience, sense of coherence, and the type of stress perceived as either threat or challenge) of the explained variables (psychological well-being in schizophrenia).

A weak yet coherent pattern of results was revealed. Stepwise regression analysis demonstrated that only manageability (a factor of sense of coherence) significantly explained hospitalization frequency: The higher manageability was, the lower the hospitalization frequency. The remaining explanatory variables (that is, factors of resilience and level of experienced stress appraised as challenge/threat) did not demonstrate significant influence on the sample.

The conducted stepwise regression analysis indicated that the SOC factors labelled as comprehensibility and manageability were significant and most prominent predictors of the following: Level of experienced mental health (level of concentration, feelings of happiness and playing a useful part, ability to face problems, make decisions and enjoy everyday activities, etc.), negative mood and level of depressiveness (negative relationship between the variables), and explaining positive mood in the sample (positive relationship). The demonstrated results may suggest that the higher comprehensibility (i.e., insight into one’s actual difficulties and mental state) is experienced by a subject, the lower their sense of happiness and joy generated in connection with the sense of mental health in the course of schizophrenia is. The third SOC factor, meaningfulness, did not prove to be a predictor for mental health or positive/negative mood. On the other hand, stress appraisal in terms of challenge or threat was demonstrated to be a significant predictor for the subjects’ mood (positive, negative and depressiveness). A higher experience of threat in stress situations was associated with a higher intensity of negative (depressive) mood.

Negative and positive symptoms, excessive arousal, and cognitive disorganization (i.e., deficits of cognitive functions) were demonstrated to be significant factors describing the level of psychological well-being in patients with schizophrenia (Table 3). An analysis of the regression models presented in Table 3 allowed the research team to identify two most frequently appearing predictors that explained schizophrenia symptoms, that is: Manageability (SOC) and resilience (RSA). The general level of resilience significantly and negatively explained the general level of schizophrenic symptoms in the sample (PANSS global index), level of negative symptoms, and cognitive disorganization in the subjects. The high level of resilience is a health-promotive resource, which may minimize the intensity of developing psychiatric symptoms. Resilience and sense of coherence as well as appraisal of the perceived stress in terms of challenge or threat were all demonstrated to be insignificant in terms of explaining the intensity of positive symptoms in the sample. Some interest can be generated by the Beta coefficients which demonstrate that, apart from resilience, comprehensibility as a dimension of SOC was also a significant predictor for psychiatric symptoms in the sample. The relationship was positive, which meant that higher comprehensibility (indicating the degree to which reality seems possible to be grasped and explained) was associated with higher disorganization of cognitive functions and excessive arousal in subjects with schizophrenia. No significant relationship was found between resilience and sense of coherence, stress appraisal in terms of challenge/threat and anxiety and depression—the Beta coefficients between these variables were insignificant.

## 4. Discussion

While the study was exploratory, its aim was to identify courses of further thorough explorations concerning predictors of the disease and treating patients with schizophrenia.

The obtained results suggest a lack of unambiguity or dominance of negative mood, depressiveness, anxiety, dysphoria, and symptoms in the subjects. The subjects were patients in the course of pharmacological treatment and psychiatric rehabilitation, which may explain these results.

The results also revealed that patients with schizophrenia demonstrated a low level of such personal resources as general resilience and sense of coherence in comparison with norms, at the same time pointing to appraising the experienced stress frequently as a threat rather than a challenge. Considering that the study entailed measuring the predictive strength of all the three mentioned personal resources simultaneously with regard to psychological well-being, which also comprised several components (psychiatric symptoms, general level of mental health and mood) in a sample of patients with schizophrenia who had been treated for many years, it is difficult to compare the results closely with those obtained by other authors—no study with a similar model comprising such a large number of study variables conducted in a sample of patients of one particular group originating from one geographic location has been found. Literature presents results of studies which indicate, similarly to the presented study, that a low level of coping with stress is associated with appraising stress as a threat and avoiding the situation passively [40,41,42]. Stress appraisal in terms of threat and passive avoidance of stress situations is a variable which was proven by some studies to be significantly related with emotional reactivity, mood and, thus, a stronger reaction to stress [42,43]. Some studies identified disorders within the ability to differentiate and recognize various emotional states and mood in patients with schizophrenia [42]. It was also pointed out that deficits of processing emotions serve as a basis for delusions and hallucinations [42]. Disorders in perceiving and interpreting emotions appear in the form of misinterpreting negative emotions rather than neutral ones [42,44]. The appraisal of stress in subjects may also be associated with the perception of disease. The mean duration of the disease in examined individuals was 10 years. Świtaj and colleagues [27] suggested that many individuals with mental illness frequently internalize mental illness stigma. This factor can be a stressor, diminishing the ability to adapt the stressful situations. In turn, the sense of coherence is the important, protective factor for well-being.

The presented studies revealed that resilience significantly predicts the intensity of psychiatric symptoms—both negative symptoms of schizophrenia (higher resilience was associated with a lower intensity of negative symptoms) and cognitive disorganization. The presented study demonstrated that while resilience significantly predicted negative symptoms, appraising stress in terms of threat demonstrated significant predictive power for negative (depressive) mood. Studies by Cooper and colleagues [45] and Hjemdal and colleagues [30], similarly to the presented research work, highlighted the relationship between mental toughness and positive emotions, emphasizing the importance of resilience as a predictor for mental health in conjunction with individual differences in tolerance for stress.

Regarding studies that identified a significant relationship between sense of coherence and psychiatric symptomatology (positive and negative symptoms), results similar to the presented ones concerning the strength of the relationship between the mentioned variables were obtained by Bengtsson-Tops et al. [46], Gassmann et al. [47], and Witkowska-Łuć [28]. It should be noted that the abovementioned study by Witkowska–Łuć did not measure the relationship between sense of coherence and positive and negative mood in patients with schizophrenia [28]. Witkowska-Łuć conducted her study in a sample of 134 patients with schizophrenia using the SOC and PANSS scales to measure the variables. Results of the study revealed a significant relationship between comprehensibility, general sense of coherence, and the intensity of psychopathology in terms of negative symptoms. Her study demonstrated that higher results in comprehensibility were associated with less severe negative symptoms at the start of hospitalization of patients with schizophrenia. However, her analysis of the relationship of the level of reduction of psychopathological symptoms over time demonstrated negative correlations. The higher results in comprehensibility were associated with the lower improvement in terms of negative symptoms in hospitalized patients. A similar relationship was observed in the presented study. Two of the SOC dimensions, manageability and meaningfulness, did not prove to be significantly related with the PANSS scores. Neither the presented study nor the research work of Witkowska-Łuć [28] revealed a significant relationship between SOC and the intensity of positive symptoms. These relationships can be interpreted as indicators of insight into the process of disease development (see [28]). The result may also suggest biological grounds for positive symptoms, which may explain the lack of relationship between positive symptoms and SOC. Bengtsson-Tops et al. [46], Gassmanna et al. [47], and Witkowska-Łuć [28] in their studies in samples of patients with schizophrenia pointed to similar relationships: Higher manageability was associated with a lower intensity of disease symptoms, subjects’ better functioning, and better course of treatment. The remaining two dimensions of SOC, comprehensibility and meaningfulness, were not related with the intensity of schizophrenic, positive symptoms. The authors of the presented study obtained similar results.

In the analysis of the relationship between SOC and mood and emotions, it is also worthwhile to quote the results of Chiu-Yueh and Yun-Fang concerning Taiwanese families, where a lower sense of coherence was associated with a higher sense of burden in female caretakers of individuals with schizophrenia remaining in ambulatory psychiatric care [48]. The presented study did not reveal a similar relationship between resilience, SOC, and anxiety and depression. In a study by Friborg and colleagues, resilience and all its components were significantly linked with the dimension of depression and anxiety [22]. The difference between the results of the study by Friborg and colleagues [22] and the presented research work may be caused by numerous factors: Different measures used, the nature of the sample or the methodological limitations of the presented study. Further studies should continue to explore relationships linking anxiety and depression with resilience and sense of coherence.

### Study Limitations

There are certain limitations to the presented study. The research plan adopted in the described research report was based on the cross-sectional study and correlation-regression research model. The measurement was carried out only once, and then, a regression analysis was performed to establish predictors for the explained variables. However, the theoretical and then methodological assumptions provide the basis for estimating the correlation and in the subsequent stage of regression and determining the relevant predictors of the variables explained. Future longitudinal studies would ensure a more extensive measurement of processes underlying the well-being in the course of schizophrenia.

The obtained results concern young adults diagnosed with schizophrenia (mean age: 29). The average duration of the disease (10.7 years) indicated a long-term course of the psychotic disorder in the sample. Thus, the analyzed level of personal resources (resilience, sense of coherence, and experienced stress) may be largely determined by the long course of the mental disorder. The results of the study can be analyzed and compared solely in the context of patients diagnosed with schizophrenia with many years of disease history. Patients whose duration of the disease was short and who had experienced a crisis caused by their first psychotic episode did not participate in the study. Quality of life was found be impaired in people with schizophrenia and associated with coping, reappraisal, social support, and duration of hospitalization [49]. Further research is required to study the relationship between sense of coherence and quality of life in people with schizophrenia. Sample inclusion was based on patients’ consent rather than random sampling. Nevertheless, the convenient sample procedure is allowed in clinical studies. The conclusions drawn require verification through studies conducted in a more diverse population of patients with schizophrenia, which is in line with the authors’ further intention.

## 5. Conclusions

The results of the presented study demonstrated the following to be significant psychological predictors of psychological well-being in patients with schizophrenia:

- Resilience (explaining significantly the level of schizophrenic symptomatology: Negative symptoms and cognitive disorders);
- sense of coherence, which significantly predicted mood (in the case of manageability) and psychiatric symptomatology (comprehensibility).

The level of stress appraised as challenge predicted positive mood, while stress appraisal in terms of threat served as a predictor for negative mood and depressiveness. The obtained results confirm that resilience, sense of coherence, and stress appraisal in terms of challenge are significant (yet not the sole) predictors of psychological well-being in patients with schizophrenia treated for years and recovering from disease. The study outcomes also suggest that, apart from resilience, comprehensibility is a vital dimension of SOC, which may be significant for the process of treating and recovery of individuals with schizophrenia. Therefore, constructing therapeutic programs and performing the long-term process of psychiatric rehabilitation of patients with schizophrenia should incorporate enhancing mental toughness, working therapeutically on correcting patients’ stress appraisal so that it shifts from threat to challenge, and fostering sense of coherence (particularly manageability) in individuals suffering from various forms of schizophrenia.

## Figures and Tables

**Table 1:** Indices of components of the sociodemographic variables and explained variable of patients suffering from schizophrenia (*N* = 201).

Psychological Well-being	M	SD
Psychiatric symptomatology	3.52	1.33
Level of mental health	2.32	0.68
Mood level		
Positive mood	3.95	1.07
Negative mood	3.91	1.18
**Sociodemographic Variables**	**% of Subjects with Schizophrenia**	
Sex (%)	Female	47.3
	Male	52.7
Age	Mean	28.97
	Range	18–62
Education completed (%)	Elementary school	8.5
	Vocational school	2.5
	High school	53.7
	University	35.3
Source of income (%)	Permanent job	10.4
	Permanent/temping job/parents/annuities	16.0
	Temping job	3.5
	Temping job/parents/scholarship	-
	Parents/other people	27.9
	Parents/annuities/social aids	14.5
	Scholarship	1.0
	Annuities	20.9
	Allowance/social aids/annuities	5.0
Living conditions (%)	With both parents/	50.7
	One parent	22.9
	With someone else	13.4
	Alone	10.9
Siblings	Only child	14.9
	One sibling	50.2
	Two siblings	19.4
	Three or more siblings	15.4
Parents’ education	Father	
	-elementary school	6.5
	-incomplete high school	14.4
	-high school	35.8
	-university	39.3
	-higher than university	2.5
	Mother	
	-elementary school	9.0
	-incomplete high school	8.5
	-high school	38.3
	-university	43.3
	-higher than university	0.5
Family economic situation	Very bad/bad	3.5
	Rather bad	9.0
	Tolerable	33.8
	Rather good	28.9
	Good/very good	24.9

Note: Psychiatric symptoms—indices measured with the PANSS; level of mental health—measured with the GHQ-12 scale; mood level—measured with the mood scale.

**Table 2:** Mean scores for components of resilience, sense of coherence, and level of perceived stress in patients with schizophrenia (*N* = 201).

Personal Resources	M	SD
Resilience	63.01	
Perception of self	17.72	5.25
Perception of future	12.72	4.37
Personal strength	30.38	8.76
Resilience (general score)	63.01	14.43
Sense of coherence Sense of coherence (general score)	122.41	26.27
Comprehensibility	42.00	10.22
Manageability	42.83	10.35
Meaningfulness	37.66	9.72
Perceived stress		
Perceived efficiency of coping with stress (general score)	8.94	3.89
Stress appraised as challenge	5.74	2.85
Stress appraised as threat	18.42	5.47

**Table 3:** Summary of the regression model for the explained research variables.

Explained Variable		Significant Predictors	Model Summary
Course of the disease	Number of hospitalizations/years of disease	n.s.	n.s.
	Number of hospitalizations	SOC Manageability Beta = −0.212	F(3144) = 2.53 R2 corrected = 0.050 *p* < 0.05
Psychological well-being	Mental health	SOC comprehensibility Beta = 0.468	F(3144) = 30.83 R2 corrected = 0.37 *p* < 0.001
	Positive mood	SOC manageability Beta = 0.580 SOS stress appraised as challenge Beta = 0.164	F(3144) = 49.23 R2 corrected = 0.496 *p* < 0.001
	Negative mood, depressiveness	SOC manageability—Beta = −0.534 SOS stress appraised as threat Beta = 0.190	F(2145) = 49.60 R2 corrected = 0.398 *p* < 0.001
Psychological well-being (Psychiatric symptoms)	General index	RSA Resilience Beta = −0.302	F(3144) = 3.08 R^2^ corrected = 0.041 *p* < 0.05
	Positive symptoms	n.s.	n.s.
	Negative symptoms	RSA Resilience Beta = −0.385 SOC Comprehensibility Beta = 0.311	F(4143) = 2.94 R2 corrected =0.050 *p* < 0.05
	Excessive arousal	SOC Comprehensibility Beta = 0.173	F(2145) = 3.97 R^2^ corrected = 0.039 *p* < 0.05
	Cognitive disorganization	SOC Comprehensibility Beta = 0.330 RSA Resilience Beta = −0.303	F(3144) = 4.16 R2 corrected = 0.060 *p* < 0.01
	Depression	n.s.	n.s.
	Anxiety	n.s.	n.s.

Note: SOC—Comprehensibility (dimension of sense of coherence); RSA—resilience; n.s.—nonsignificant.
